# Identifying biomarkers for papilledema and pseudopapilledema

**DOI:** 10.1038/s41598-025-09778-2

**Published:** 2025-07-10

**Authors:** Rishi Sekhri, Helen J. Kuht, Zhanhan Tu, Gail D. E. Maconachie, Riddhi Shenoy, Esha Prakash, Seema Teli, Sohaib R. Rufai, Nagini Sarvananthan, Tahir Islam, Michael Hisaund, Rebecca J. McLean, Indranil Choudhuri, Joris Dehaene, Martin Barnes, Irene Gottlob, Ian DeSilva, Mervyn G. Thomas

**Affiliations:** 1 Ulverscroft Eye Unit, School of Psychology and Vision Sciences, College of Life Sciences, University of Leicester, Leicester, UK; 2 School of Allied Health Professions, Nursing and Midwifery, Faculty of Health, University of Sheffield, Sheffield, UK; 3 Department of Ophthalmology, Leicester Royal Infirmary, Leicester, University Hospitals of Leicester NHS Trust, Leicester, UK; 4 Department of Neurology, Cooper University Hospital, Camden, NJ USA; 5 NIHR Leicester Biomedical Research Centre, University Hospitals of Leicester NHS Trust and University of Leicester, Leicester, UK

**Keywords:** Papilledema, Pseudopapilledema, Optical coherence tomography, Optic disc drusen, Tilted optic disc, Crowded optic disc, Diagnostic markers, Optic nerve diseases

## Abstract

Papilledema describes optic nerve head (ONH) swelling due to raised intracranial pressure which can lead to complications including visual loss. Differentiating it from pseudopapilledema is often challenging and may require invasive investigations. Optical Coherence Tomography (OCT) is a non-invasive imaging modality, allowing visualisation of the ONH. We identified OCT-derived biomarkers for papilledema and pseudopapilledema in the existing literature. 213 patients with confirmed papilledema, optic disc drusen (ODD), tilted optic discs (TOD) or crowded optic discs (COD) were included. OCT scans were analysed for qualitative and quantitative biomarkers, with sensitivity, specificity, and AUC calculated for individual and combined biomarkers. Logistic regression modelling including qualitative biomarkers to differentiate papilledema from ODD and COD demonstrated a sensitivity and specificity of 89% and AUC of 0.96. Inclusion of TOD reduced the sensitivity of the model to 66%. In a model differentiating papilledema from TOD, the best-performing biomarker achieved a sensitivity of 87%, specificity of 61% and AUC of 0.83. Using qualitative biomarkers, we identified a model with high sensitivity and specificity to differentiate papilledema from ODD and COD. Quantitative biomarkers displayed high AUCs for differentiating TOD from papilledema. Our findings show that OCT demonstrates promising utility in differentiating papilledema from pseudopapilledema.

**Supplementary Information:**

The online version contains supplementary material available at 10.1038/s41598-025-09778-2.

## Introduction

Papilledema describes the swelling of the optic nerve head (ONH) due to raised intracranial pressure (ICP) which can lead to significant complications such as visual loss and brain herniation, which may result in death^1,2^. Pseudopapilledema arises from a group of usually benign conditions, including optic disc drusen (ODD), tilted optic discs (TOD) and crowded optic discs (COD), that may visually mimic papilledema, but are not associated with raised ICP or true swelling of the ONH. A range of different investigative modalities are used to complement the clinical history and examination to differentiate papilledema from pseudopapilledema^3^. In scenarios where ODD are visible or in severe grades of papilledema, it may be possible to obtain a clinical diagnosis from direct ophthalmoscopy. However, a significant proportion of cases pose a diagnostic challenge, as they present either with lower grades of papilledema or “softer” signs of pseudopapilledema. Therefore, it is not uncommon for individuals with suspected papilledema to undergo expedited invasive and expensive investigations including neuroimaging and lumbar puncture due to the sight and life-threatening nature of papilledema^1^. In such scenarios, differentiating between sight or life-threatening papilledema and benign pseudopapilledema presents an important diagnostic challenge.

Previous studies have highlighted the role of B-scan ultrasound^4,5^, fundus autofluorescence^6–8^ and fluorescein angiography^6^, However, these investigations have clear limitations such as reduced sensitivity for detecting drusen depending on the calcification or location of drusen in the ONH. Additionally, whilst fluorescein angiography has shown promise in differentiating papilledema from pseudopapilledema^9^, it is both invasive and time-consuming, thus limiting its use in everyday clinical practice. In recent years, optical coherence tomography (OCT) has emerged as the modality of choice for detecting ODD and is almost considered an extension of the routine ophthalmological examination^10^. OCT is non-invasive, demonstrates a higher detection rate of buried ODD than B-scan ultrasound^10,11^, provides detailed information about the ONH structure, and is widely available^12,13^. The information that OCT provides has lent itself to the discovery of potential biomarkers that have been described in the literature^3,12^. We hypothesise that these biomarkers may demonstrate diagnostic utility in differentiating papilledema from pseudopapilledema.

There is an unmet need for a rapid and non-invasive test to differentiate papilledema from pseudopapilledema, which has not yet been assessed with a comprehensive set of OCT biomarkers. Moreover, previous studies have used control populations with no ONH abnormalities and have only aimed to distinguish papilledema from ODD, limiting their applicability to real-world clinical practice. In this study, we evaluate the diagnostic accuracy of a set of OCT biomarkers to determine if spectral domain OCT can reliably differentiate papilledema from pseudopapilledema in a clinical setting.

## Methods

In this retrospective cohort study, we reviewed all patients who presented with suspected papilledema or pseudopapilledema between 1st February 2021 and 31st January 2022 who attended eye casualty at Leicester Royal Infirmary. Since most patients have follow-up and further investigations within the neuro-ophthalmology service and/or ultrasound B-scan clinics we also included any additional patients from these clinical services. Using electronic admissions records, we recorded the reason for attendance and suspected diagnosis. These included entries such as “blurred margins,” “swollen optic disc,” and other descriptors suggestive of ONH abnormalities. Cases were then cross-referenced with the Heidelberg Eye Explorer database to identify those with both volume and circular OCT scans. Each diagnosis was made by an ophthalmologist and subsequently validated by members of the research study team through review of electronic health records, including clinician assessments, imaging, and ancillary investigations.

Inclusion criteria required a confirmed diagnosis of one of four conditions: papilledema (diagnosed based on raised ICP confirmed via neuroimaging or lumbar puncture), ODD (confirmed by B-scan ultrasound), COD (clinically defined as small discs with a cup-to-disc ratio < 0.1), TOD (defined by D-shaped or vertically oblique discs with asymmetrical elevation). Patients also had to have suitable OCT imaging (volume and circular scans) available at the time of diagnosis.

Exclusion criteria included patients without confirmatory testing (e.g., no lumbar puncture or B-scan ultrasound where appropriate), diagnoses other than the four conditions studied (e.g., anterior ischaemic optic neuropathy, papillitis, optic atrophy), dual pathology, unclear diagnosis at the time of assessment, or absence of OCT scans during the relevant clinical episode.

All data in this study was retrospectively collected. This study is a retrospective analysis of anonymised datasets. The study was reviewed and approved by the East Midlands—Leicester Central Research Ethics Committee (Ethics Reference: 261121). In accordance with Health Research Authority guidelines, the requirement for informed consent was waived, as all data were de-identified at source. This study adhered to the tenets of the Declaration of Helsinki.

### Literature review

A literature review was performed using OVID Medline. Results are accurate up to October 2023.

The question: “Can OCT biomarkers differentiate papilledema from pseudopapilledema?” was identified. Search terms included “papilledema”, “pseudopapilledema”, “optic disc drusen”, and “OCT”. A full list of search terms can be seen in Supplementary Table S1 online. A limit was set to the English language. 79 papers were identified with 16 of these being review articles. 34 of these papers were studies which investigated papilledema or pseudopapilledema OCT biomarkers. All other papers were case reports, replies and comments, or did not compare papilledema and pseudopapilledema OCT biomarkers. Breakdown of the literature review search results can be seen in Supplementary Figure S1 online. Of the 34 papers which detailed relevant biomarkers, the following biomarkers were identified: the presence of folds^14,15^, the presence of hypo-reflective cores with hyperreflective caps^16–18^, the presence of hyperreflective lines^17^, Bruch’s membrane/retinal pigment epithelium (BM/RPE) shape^19^, Bruch’s membrane opening (BMO) diameter^16,17,20–22^, the presence of peripapillary hyperreflective ovoid mass-like structures (PHOMS)^17,23–26^, maximum papillary height^16,22,27^, and retinal thickness measurements, including retinal nerve fibre layer (RNFL) thickness^16,17,19–22,27–44^. PHOMS were included as a biomarker rather than as a distinct category a priori, based on previous studies that have demonstrated their frequent presence across a broad spectrum of clinical diagnoses, including both papilledema and pseudopapilledema^10,26^. Other OCT biomarkers that were identified in the literature review but not included in this study include OCT angiography biomarkers^34,45^, alpha angle^40^, ONH volume^32^, and custom quantitative parameters^33^. Additionally, biomarkers detected by artificial intelligence algorithms were not included in this study^46^. Previous biomarkers that involved detection of a subretinal hypo-reflective space^40,43^ were excluded as these are now thought to be artefacts associated with poor penetrance of previous OCT technologies^47,48^.

### OCT analysis

**Fig. 1:**
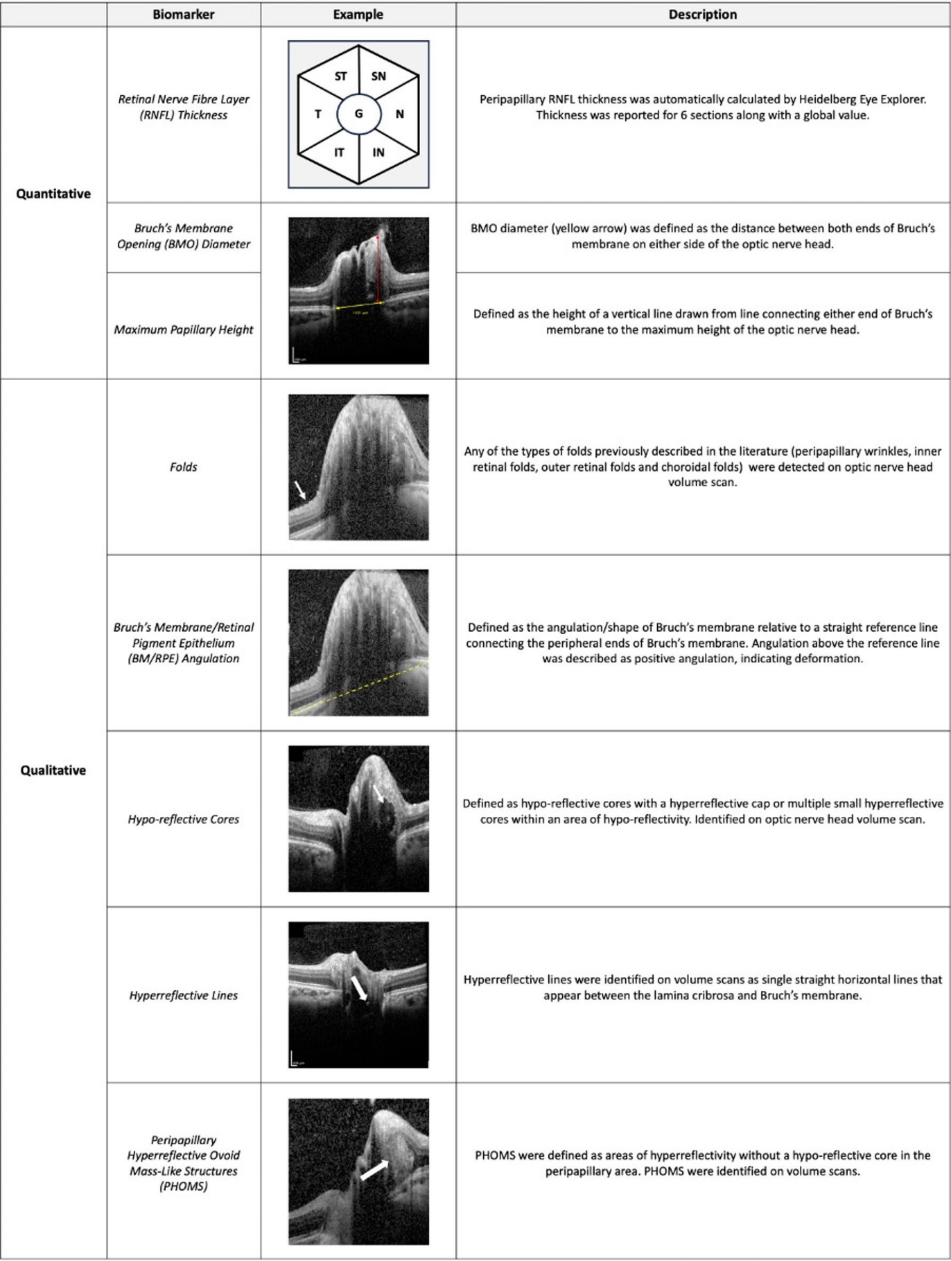
Descriptions and examples of all quantitative and qualitative biomarkers included in this study. SN, superonasal; N, nasal; IN, inferonasal; IT, inferotemporal; T, temporal; ST, superotemporal; G, global; RNFL, retinal nerve fibre layer; BMO, Bruch’s membrane opening; BM/RPE, Bruch’s membrane/retinal pigment epithelium; PHOMS, peripapillary hyperreflective ovoid mass-like structures.

OCT scans were analysed on Heidelberg Eye Explorer (Heidelberg Engineering, Heidelberg, Germany). Both circular and volume scans were analysed for each eye. Scans obtained using Enhanced Depth Imaging (EDI) were used when available. For the ONH volume scan, the device was set to a 15° × 15° rectangle centred on the optic disc. This rectangle contained 73 B-scans. The circular peripapillary scan was set to a 3.4mm circle around the optic disc. The ONH volume scan was used to assess all biomarkers except for peripapillary RNFL thickness which was assessed using the circular peripapillary scan. Biomarkers were split into two groups, qualitative and quantitative. The full list of biomarkers, examples, and descriptions can be found in Fig. 1.

For quantitative biomarkers, the following criteria were used: RNFL was automatically calculated by Heidelberg Eye Explorer. Thicknesses of 6 segments were reported along with a global value. The circular scans that this data was derived from were manually reviewed for segmentation errors and corrected where appropriate. BMO diameter was defined as the horizontal distance between both ends of Bruch’s membrane on either side of the ONH, at the level of the BM/RPE^22^. This was measured on the B-scan which was located at the widest part horizontally of the ONH using the caliper tool on Heidelberg Eye Explorer. Maximum papillary height was defined as the vertical distance between the centre of a line connecting both ends of Bruch’s membrane and the maximum height of the ONH^22^. This was measured using the caliper tool on Heidelberg Eye Explorer. For qualitative biomarkers, the following criteria were used: folds were identified in accordance with previous literature^12,15^. Any of the four types of folds (peripapillary wrinkles, inner retinal folds, outer retinal folds and choroidal folds) were reported generically as folds^15^. Folds were identified on ONH volume scans only. BM/RPE angulation was identified relative to a straight reference line connecting the peripheral ends of Bruch’s membrane on the B-scan which intersected most central to the ONH^12^. This was assessed on the volume scan. Angulation above the reference line was reported as positive angulation. Hypo-reflective cores required a hyperreflective cap or small hyperreflective cores within an area of hypo-reflectivity, as described by the Optic Disc Drusen Studies Consortium^10^. The location of identified hypo-reflective cores were cross-referenced with IR fundus images to avoid mistaking blood vessels for hypo-reflective cores. Hyperreflective lines were identified as straight horizontal lines that appeared between the lamina cribrosa and Bruch’s membrane^3^. These lines were distinguished from the caps of hypo-reflective cores by checking adjacent B-scans^49^. PHOMS were identified as areas of hyperreflectivity without any hypo-reflective cores in the peripapillary area^10,26^. Hypo-reflective cores, hyperreflective lines and PHOMS were detected on ONH volume scans.

### Statistical analysis

All statistical analysis was performed using STATA (16.1, StataCorp LLC, College Station, TX). All analyses were considered statistically significant when a probability value of *p* ≤ 0.05 was identified. To account for inter-eye correlation, a per-subject unit of analysis was used (i.e., for continuous data, an average of both eyes was calculated, and for binary data, a subject was included if either eye had the biomarker present). Normality testing of the continuous variables’ distribution was carried out using the Shapiro-Wilks test. The Kruskal–Wallis test was used to test for a statistical difference in medians between the ages of patients, BMO diameter, RNFL thicknesses and maximum papillary height for each diagnostic group. Bonferroni correction was applied for the quantitative biomarkers during subgroup analysis with adjusted P values reported to control for type 1 error. The differences in proportions of patients with or without qualitative biomarkers and the differences in proportions of male to female for each diagnostic group were measured using Pearson’s chi-squared test.

Receiver operating characteristic (ROC) curves were plotted and areas under the curve (AUC) were calculated for individual quantitative biomarkers. Sensitivity and specificity for detecting papilledema were then calculated for a selected cut-off for the biomarker which displayed the highest AUC, as this model would demonstrate the best measure as a discriminator.

Logistic regression models were created for both individual and combinations of qualitative biomarkers. The sensitivity and specificity of each individual biomarker and overall model including multiple qualitative biomarkers for detecting papilledema were calculated. A ROC curve was then created for this overall model and AUC was calculated.

The sensitivity and specificity of both the set of qualitative and quantitative biomarkers were calculated for the detection of papilledema in the overall group including all cases of papilledema and pseudopapilledema. The sensitivity, specificity and AUCs of the biomarkers, and models for detecting papilledema were then calculated for the following subgroups: papilledema with ODD and COD, and papilledema with TOD.

### Meeting presentation

Presented at the Association for Research in Vision and Ophthalmology Annual Meeting, 2023, New Orleans, USA.

## Results

### Demographics

From 470 patients initially identified, 257 were excluded based on predefined eligibility criteria, resulting in a final cohort of 213 patients with confirmed diagnoses and suitable OCT imaging. These included 79 patients with papilledema (37.1%), 76 patients with ODD (35.7%), 44 patients with TOD (20.7%), and 14 patients with COD (6.6%). The median age of the cohort was 22 (aged 5–76 years, IQR = 19). The cohort included 153 females (71.8%) and 60 males (28.2%). The median ages of the patients in each diagnostic group were 26 in the papilledema group (aged 5–61 years, IQR = 19), 17 in the ODD group (aged 8–76 years, IQR = 18), 18.5 in the TOD group (aged 6–58 years, IQR = 17.5) and 12 in the COD group (aged 6–41 years, IQR = 24) (p = 0.008). The papilledema group included 68 females (86.1%), the ODD group included 49 females (64.5%), the TOD group included 25 females (56.8%), and the COD group included 11 females (78.6%) (p = 0.002).

### Quantitative biomarkers

**Fig. 2:**
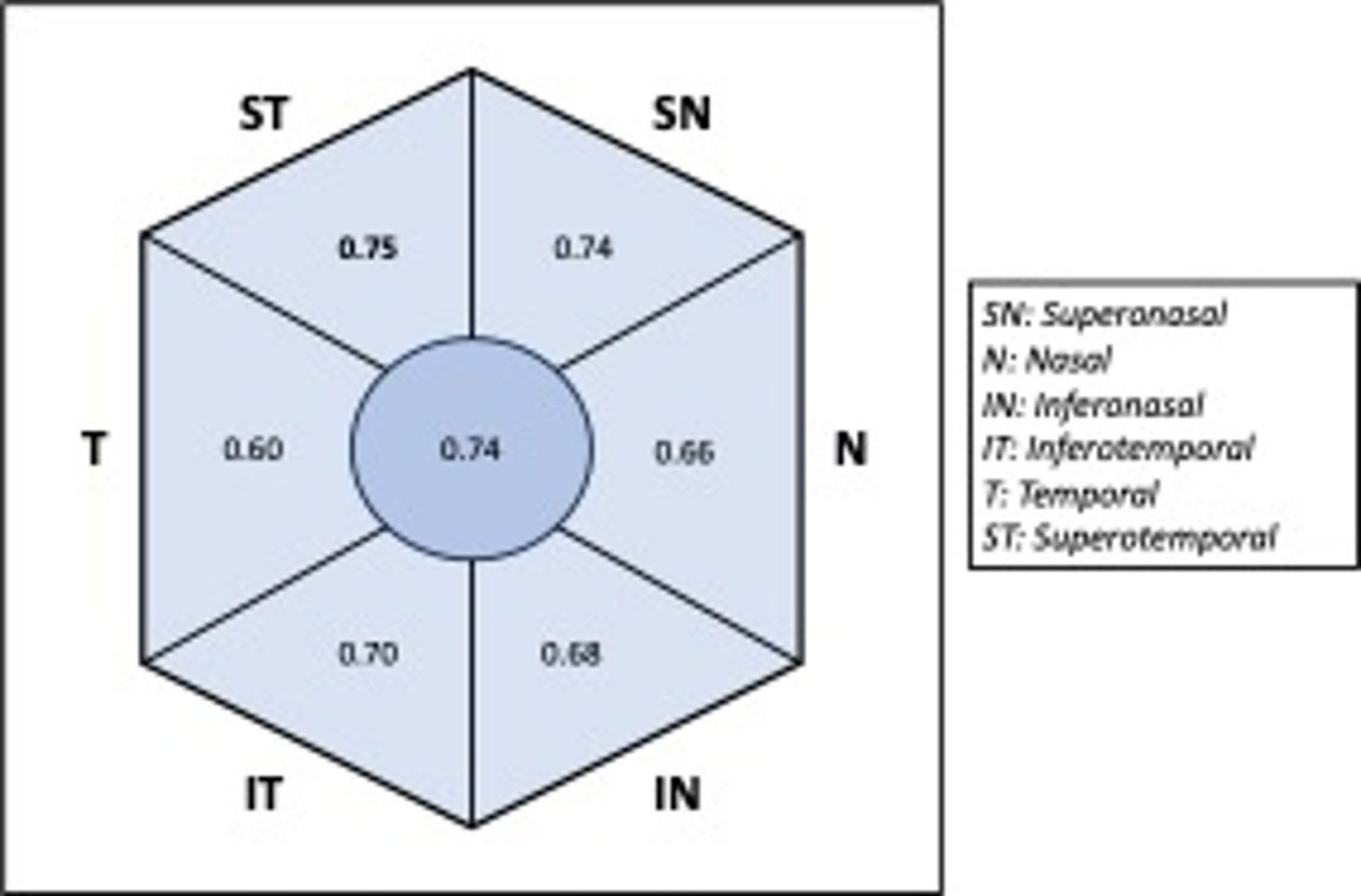
Diagram illustrating optic nerve head segments for retinal nerve fibre layer analysis and corresponding area under curve values for each segment in overall cohort. SN, superonasal; N, nasal; IN, inferonasal; IT, inferotemporal; T, temporal; ST, superotemporal.

**Fig. 3:**
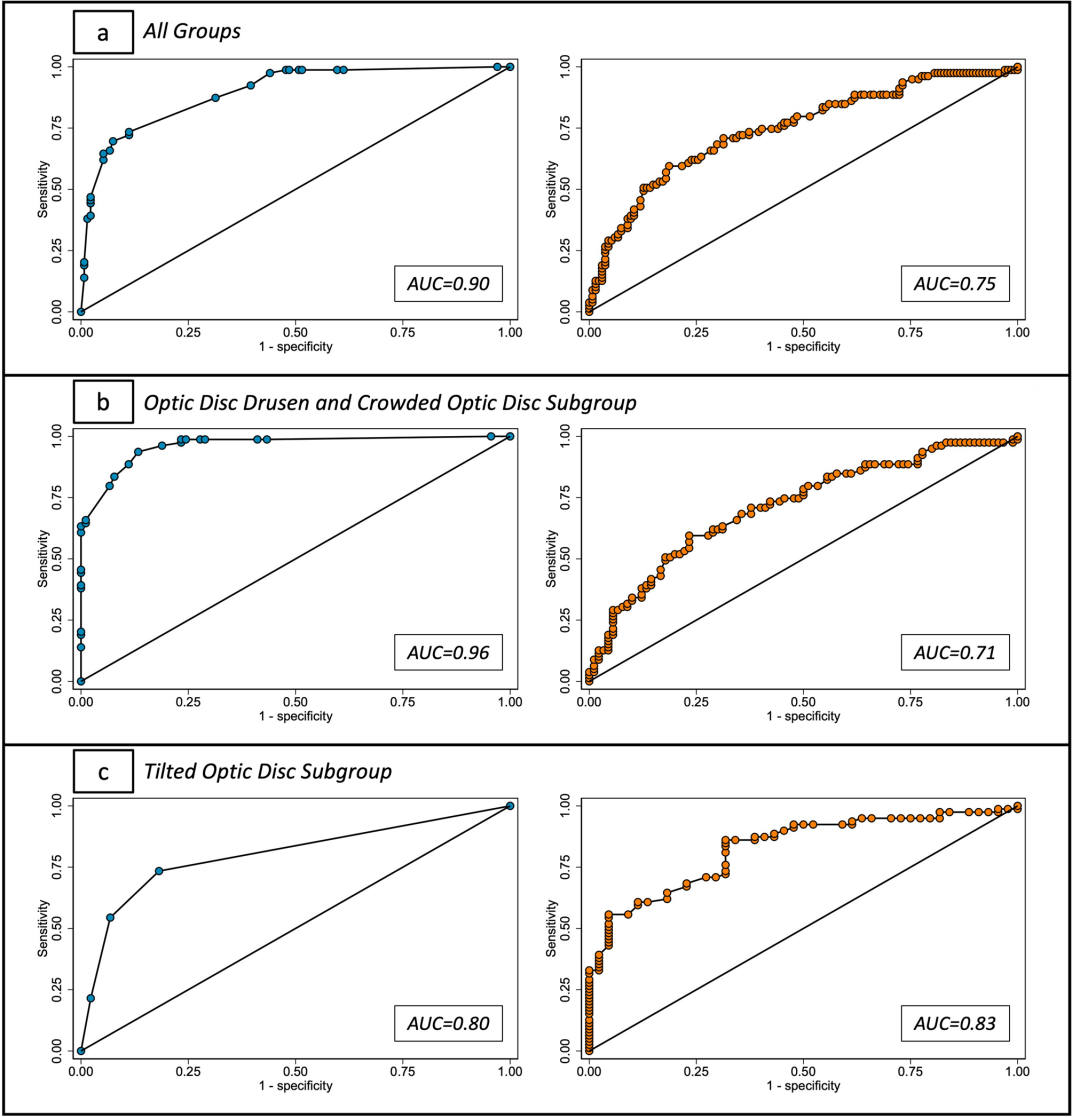
ROC curves for both qualitative biomarkers (blue curve) and quantitative biomarker with highest AUC (orange curve) in overall group (**a**), ODD and COD subgroup (**b**), and TOD subgroup (**c**). ROC, receiver operating characteristic; AUC, area under curve; ODD, optic disc drusen; COD, crowded optic disc; TOD, tilted optic disc.

There were significant differences between all four diagnostic groups for all quantitative biomarkers except for temporal RNFL thickness (*p* = 0.818). AUCs of the ROC curves for quantitative biomarkers in this study ranged from 0.60 for temporal RNFL thickness to 0.75 for superotemporal RNFL thickness (Table 1) (Figs. 2, 3a). When using a cut-off value of 128µm for superotemporal RNFL thickness, a sensitivity of 89.9% and specificity of 27.6% was achieved for distinguishing between papilledema and the other diagnostic groups.

**Table 1:** Median values, *P* values, and AUC values for each quantitative biomarker in each diagnostic category in overall cohort.

		Diagnosis					
Biomarker		Pap (n = 79)	ODD (n = 76)	TOD (n = 44)	COD (n = 14)	*P* value	AUC
Global RNFL (µm)	Median (IQR)	122.5 (48)	107.3 (29.5)	93.8 (20.5)	104.8 (18.5)	< 0.001	0.74
Superonasal RNFL (µm)	Median (IQR)	156.5 (78.5)	114.8 (52.8)	103.3 (35.5)	112.3 (51)	< 0.001	0.74
Nasal RNFL (µm)	Median (IQR)	85 (50.5)	79.5 (36.5)	64.8 (26.8)	76.8 (24.5)	< 0.001	0.66
Inferonasal RNFL (µm)	Median (IQR)	147 (84)	125.3 (44.3)	102.3 (44.8)	128 (54.5)	< 0.001	0.68
Inferotemporal RNFL (µm)	Median (IQR)	170 (58)	152.3 (34.5)	146.8 (25.3)	146.3 (31)	< 0.001	0.70
Superotemporal RNFL (µm)	Median (IQR)	173 (59)	142.3 (39.5)	141.5 (33.5)	150.8 (25.5)	< 0.001	0.75
Temporal RNFL (µm)	Median (IQR)	79 (18)	74 (17.5)	76.3 (18.5)	73.3 (9)	0.818	0.60
BMO diameter (µm)	Median (IQR)	1446.5 (197)	1366.5 (155.5)	1440.8 (207)	1361.8 (186)	0.006	0.61
Maximum papillary height (µm)	Median (IQR)	746.5 (134)	721.8 (178.5)	624.5 (154.5)	698 (131.5)	< 0.001	0.66

BMO, Bruch’s membrane opening; RNFL, retinal nerve fibre layer; ODD, optic disc drusen; TOD, tilted optic discs; COD, crowded optic discs; AUC, area under curve; IQR, interquartile range.

### Qualitative biomarkers

There were significant differences in the prevalence of biomarkers between all 4 diagnostic groups (*p* =  < 0.001–0.024). Sensitivities of biomarkers for distinguishing between papilledema and the other diagnostic groups ranged from 40.5 to 89.9%, with BM/RPE shape and the absence of hypo-reflective cores displaying the lowest and highest sensitivities, respectively. Specificities of biomarkers for distinguishing between papilledema and the other diagnostic groups ranged from 17.9 to 93.3%, with the presence PHOMS and the presence of folds displaying the lowest and highest specificities, respectively. A logistic regression model, including all qualitative biomarkers, achieved an overall sensitivity of 65.8% and specificity of 93.3% for distinguishing papilledema from all other diagnostic groups (Table 2). The ROC curve for this logistic regression model displayed an AUC of 0.90 (Fig. 3a).

**Table 2:** Prevalence of each qualitative biomarker in each diagnostic category in overall cohort.

		Diagnosis									
Biomarker		Pap (n = 79)	ODD (n = 76)	TOD (n = 44)	COD (n = 14)	Odds ratio	*P* value	Sensitivity		Specificity	
Hypo-reflective Cores (absence)	N (%)	71 (89.9)	3 (4.0)	43 (97.7)	12 (85.7)	15.04	< 0.001	89.87	65.8	56.72	93.3
Hyperreflective Lines (absence)	N (%)	62 (78.5)	13 (17.1)	37 (84.1)	10 (71.4)	1.28	< 0.001	78.48		55.22	
PHOMS (presence)	N (%)	65 (82.3)	68 (89.5)	34 (77.3)	8 (57.1)	1.20	0.024	82.28		17.91	
BM/RPE Angulation (+ve)	N (%)	32 (40.5)	5 (6.6)	6 (13.6)	1 (7.1)	4.68	< 0.001	40.51		91.04	
Folds (presence)	N (%)	43 (54.4)	6 (7.9)	3 (6.8)	0 (0)	29.94	< 0.001	54.43		93.28	

*P* values, sensitivity, and specificity reported for each individual biomarker. Logistic regression odds ratios for each biomarker and overall sensitivity and specificity reported.

BM/RPE, Bruch’s membrane/retinal pigment epithelium; PHOMS, peripapillary hyperreflective ovoid mass-like structures; Pap, papilledema; ODD, optic disc drusen; TOD, tilted optic disc; COD, crowded optic disc.

### Subgroup: ODD and COD

A subgroup analysis excluding TOD, and therefore comparing papilledema to ODD and COD, included a total of 169 patients.

In this subgroup, all quantitative biomarkers displayed a significant difference between all three subgroups except for RNFL thickness in the nasal region (*p* = 0.061). AUCs of the ROC curves for quantitative biomarkers ranged from 0.60 for maximum papillary height to 0.71 for superotemporal RNFL thickness (Table 3) (Fig. 3b). When using a cut-off of 128 µm for superotemporal RNFL thickness, a sensitivity of 90.0% and specificity of 23.3% were achieved.

**Table 3:** *P* values and AUCs reported for all quantitative biomarkers in the optic disc drusen and crowded optic disc subgroup analysis.

Biomarker	*P* Value	AUC
Global RNFL (µm)	< 0.001	0.70
Superonasal RNFL (µm)	< 0.001	0.70
Nasal RNFL (µm)	0.061	0.61
Inferonasal RNFL (µm)	0.020	0.63
Inferotemporal RNFL (µm)	< 0.001	0.69
Superotemporal RNFL (µm)	< 0.001	0.71
Temporal RNFL (µm)	0.039	0.61
BMO diameter (µm)	0.002	0.65
Maximum papillary height (µm)	0.021	0.60

BMO, Bruch’s membrane opening; RNFL, retinal nerve fibre layer; AUC, area under curve.

There were significant differences in the prevalence of each qualitative biomarker in this subgroup (*p* =  < 0.001–0.011). Sensitivities of individual biomarkers for diagnosing papilledema in this subgroup ranged from 40.5 to 89.9%. Specificities ranged from 15.6 to 93.3%. In a logistic regression model, including all quantitative biomarkers, a sensitivity of 88.6% and a specificity of 88.9% were achieved (Table 4). The ROC curve of this model displayed an AUC of 0.96 (Fig. 3b).

**Table 4:** *P* values, sensitivity, and specificity reported for each individual quantitative biomarker in the optic disc drusen and crowded optic disc subgroup analysis.

Biomarker	Odds ratio	*P* value	Sensitivity		Specificity	
Hypo-reflective Core (absence)	126.77	< 0.001	89.87	88.6	83.33	88.9
Hyperreflective Lines (absence)	2.90	< 0.001	78.48		74.44	
PHOMS (presence)	1.78	0.011	82.28		15.56	
BM/RPE Angulation (+ve)	6.71	< 0.001	40.51		93.33	
Folds (presence)	115.09	< 0.001	54.43		93.33	

Logistic regression odds ratios for each biomarker and overall sensitivity and specificity reported.

PHOMS, peripapillary hyperreflective ovoid mass-like structures; BM/RPE, Bruch’s membrane/retinal pigment epithelium.

### Subgroup: TOD

A subgroup analysis excluding both ODD and COD compared papilledema to TOD only. 123 patients were included in this subgroup.

All quantitative biomarkers, except for temporal RNFL thickness (*p* = 0.39) and BMO diameter (*p* = 1.00), displayed significant differences between the two diagnostic groups. ROC curves for quantitative biomarkers in this subgroup displayed AUCs ranging from 0.53 for BMO diameter to 0.83 for global RNFL thickness (Table 5) (Fig. 3c). When using a cut-off of 99.5µm for global RNFL thickness, a sensitivity and specificity of 87.3% and 61.4% were achieved, respectively.

**Table 5:** *P* values and AUCs reported for all quantitative biomarkers in the tilted optic disc subgroup analysis.

Biomarker	*P* Value	AUC
Global RNFL (µm)	< 0.001	0.83
Superonasal RNFL (µm)	< 0.001	0.82
Nasal RNFL (µm)	< 0.001	0.75
Inferonasal RNFL (µm)	< 0.001	0.78
Inferotemporal RNFL (µm)	< 0.001	0.73
Superotemporal RNFL (µm)	< 0.001	0.81
Temporal RNFL (µm)	0.386	0.58
BMO diameter (µm)	1.00	0.53
Maximum papillary height (µm)	< 0.001	0.79

BMO, Bruch’s membrane opening; RNFL, retinal nerve fibre layer; AUC, area under curve.

The only biomarkers that displayed significant differences in the prevalence proportions between diagnostic groups were the presence of folds (*p* =  < 0.001) and abnormal BM/RPE shape (*p* = 0.002). The absence of hypo-reflective cores (*p* = 0.11), hyperreflective lines (p = 0.45) and the presence of PHOMS (*p* = 0.50) displayed no significant differences between diagnostic groups. Sensitivities of individual biomarkers in this subgroup ranged from 40.5 to 89.9%, with the absence of hypo-reflective cores displaying the highest sensitivity. Specificities ranged from 2.3 to 93.2%, with the presence of folds displaying the highest specificity. A logistic regression model, including the presence of folds and abnormal BM/RPE shape, achieved a sensitivity of 73.4% and specificity of 81.8% for diagnosing papilledema (Supplementary Table S2 online). The ROC curve of this model displayed an AUC of 0.80 (Fig. 3c).

## Discussion

This retrospective cohort study demonstrates that a comprehensive set of OCT biomarkers can demonstrate diagnostic utility in differentiating papilledema from pseudopapilledema. We identified a model using qualitative biomarkers that could distinguish papilledema from ODD and TOD with a high sensitivity and specificity. In a separate model, using quantitative biomarkers, we were able to achieve a high AUC.

In our group comparing papilledema to all pseudopapilledema cases, both quantitative and qualitative biomarkers displayed limited clinical utility. We could not identify a suitable cut-off for quantitative biomarkers that achieved a suitable sensitivity. Qualitative biomarkers also displayed a low sensitivity for detecting papilledema in this group. After excluding TOD from the pseudopapilledema group, qualitative biomarkers displayed an increased sensitivity and specificity for detecting papilledema, however, quantitative biomarkers continued to demonstrate limited utility. When comparing papilledema to TOD only, quantitative biomarkers displayed an increased AUC with a higher specificity for detecting papilledema. Overall, this suggests that qualitative OCT biomarkers display most clinical utility when differentiating papilledema from COD and ODD but may also be useful when combined with quantitative OCT biomarkers for differentiating papilledema from TOD.

The difference in the prevalence of biomarkers between diagnostic groups indicates that there is heterogeneity in the pseudopapilledema group. This demonstrates the importance of dividing the overall pseudopapilledema group into subgroups such as TOD, COD and ODD. Previous studies have investigated the utility of OCT biomarkers on differentiating papilledema from specific subtypes of pseudopapilledema such as ODD, however, there are a lack of studies applying a comprehensive set of OCT biomarkers to a heterogeneous group of pseudopapilledema cases which is a true reflection of presentations to eye casualty and neuro-ophthalmology clinics. The real-world clinical value of previous studies may be limited as pseudopapilledema can have multiple aetiologies, such as are included in this study. Additionally, all OCT biomarkers in this study were available on a commercially available OCT system, thus allowing the findings in this study to be readily applied to clinical practice.

Previous literature suggests that RNFL thickness and BMO diameter increase with papilledema. A study by Thompson et al. (2018) found AUCs of 0.81 and 0.97 for BMO diameter and average RNFL thickness, respectively^22^. All patients in this study had pseudopapilledema due to ODD. Comparatively, when excluding our TOD group, we found lower AUCs for these biomarkers in our study. As the aforementioned study was conducted on a paediatric cohort, a possible explanation for the discrepancy between our findings and theirs could be explained by the older age of our cohort. In our study, BMO diameter displayed a low AUC in all subgroups. As we did not have access to EDI-OCT for all patients in our study, this may have meant that BMO was obscured by superficial overlying ODD, thus leading to the overestimation of BMO diameter in the ODD group. EDI-OCT has the advantage over non-EDI-OCT by allowing for delineation of the posterior border of ODD and reducing shadowing^3^. This may have been less of an issue in the aforementioned study as their cohort was younger and therefore more likely to have buried ODD which would not cause such obscuration. Despite this, when COD and ODD were excluded, BMO continued to display a low AUC in the TOD subgroup, which cannot be explained by this phenomenon. A study by Kaplan et al. (2023), also investigating quantitative biomarkers in a paediatric population demonstrated an AUC of 0.63 for BMO diameter which is more in line with the AUC achieved in our ODD and COD subgroup^20^. The authors of this study concluded that this biomarker demonstrated limited clinical value. Whilst we measured BMO diameter, a study conducted on a non-paediatric cohort by Fard et al. (2019) found no difference in BMO area between papilledema and pseudopapilledema patients^21^.

Another study by Chiu et al. (2021) found that AUCs for RNFL thickness ranged from 0.81 to 0.90, with average and superior RNFL thickness having the highest AUC^17^. This study was also conducted on a paediatric cohort and included papilledema and ODD cases only. This study found higher AUCs for RNFL thickness than we did in our study, however, this could possibly be explained by our inclusion of COD alongside ODD in our subgroup. Despite the higher AUCs demonstrated in this study, the authors could also not find a suitable cut-off for RNFL thickness, finding a high overlap in the ranges of values in both papilledema and ODD groups. The authors of this paper came to a similar conclusion as us, stating that no quantitative biomarkers showed an acceptable diagnostic accuracy in differentiating papilledema from ODD.

Whilst we have shown that RNFL thickness may not be able to successfully differentiate papilledema from pseudopapilledema, a recent study by Flowers et al. (2021) showed that RNFL thickness variability between adjacent clock-hour segments was higher in papilledema patients than in pseudopapilledema patients^31^. In this study, they were able to demonstrate an AUC of 0.98. Whilst we were not able to view clock-hour segments in our study, the results shown here are promising and future studies may build on this finding. Additionally, a recent study by Girard et al. (2022) has demonstrated AUCs of 0.99 in differentiating papilledema from ODD using artificial intelligence with ODD and prelaminar volume measurements^46^.

Previous literature suggests that both the presence of folds and abnormal BM/RPE angulation indicate raised ICP^15,50^ and a diagnosis of papilledema. Interestingly we observe them in patients without papilledema in this study. Similarly, hypo-reflective cores with hyperreflective caps directly represent ODD, making it unexpected that less than 100% of ODD cases exhibited this biomarker. While hypo-reflective cores with hyperreflective caps define the presence of ODD, not all patients with a B-scan ultrasound confirmed diagnosis demonstrated this feature in our study. This may be due to the absence of EDI scans for all patients in our study which is known to enhance the detection of ODD^11^. Moreover, studies have demonstrated cases where ODD is visible on B-scan ultrasound but not detectable with EDI-OCT, possibly due to shadowing artefacts or vessel interference^51,52^. Hyperreflective lines and hypo-reflective cores with hyperreflective caps were noted in the papilledema group. The presence of these biomarkers in the papilledema group may suggest that some patients had both papilledema and underlying ODD and were diagnosed and investigated for papilledema but not for ODD. Dual diagnoses of papilledema and ODD are not uncommon and are reported in both paediatric and adult cohorts^53,54^.

Our findings show that both the presence of folds and abnormal angulation of BM/RPE are highly specific for papilledema but not sensitive. A recent study by Reggie et al. (2021) describes similar findings, concluding that folds may help distinguish papilledema from pseudopapilledema^14^. Previous studies suggest that approximately one-third of papilledema patients have a normal BM/RPE shape^12^. In our study, 59.5% of patients with papilledema had a normal BM/RPE shape. This discrepancy may be due to the limited area covered by our ONH volume scan not picking up the unaltered area of the BM/RPE. In a review by Sibony et al.^12^ it was suggested that a 30° transverse axial scan should be used, in our study we had access to a 15° volume scan for all patients, thus the prevalence of abnormal BM/RPE shape in papilledema patients in our study may be less than expected.

We identified PHOMS in 82% of our overall cohort, with 82% and 90% of patients with papilledema and ODD demonstrating PHOMS, respectively. These numbers are largely reflected in the literature with studies finding similar prevalences of PHOMS in their study populations, even when using EDI settings^23,55,56^. PHOMS are thought to be a nonspecific OCT marker of axoplasmic stasis in the ONH^57^. As our study shows, PHOMS displayed a low specificity for papilledema. This finding backs up previous research which states that PHOMS cannot be used to differentiate papilledema from pseudopapilledema as they are nonspecific and may be present in both conditions. Interestingly, recent studies have proposed that PHOMS may be an independent and common cause of pseudopapilledema^23,58^.

ODD were confirmed using B-scan ultrasound, which, while historically considered a standard diagnostic tool, may fail to detect buried or less calcified drusen. EDI-OCT has shown higher sensitivity in some cases, particularly for less calcified drusen. As such, it is possible that some cases of ODD were missed in our cohort. However, B-scan ultrasound was performed consistently in all ODD cases at our institution, providing diagnostic uniformity across the cohort. While our study provides real-world evidence on comparisons of previously reported biomarkers, there are some limitations due to the unavailability of EDI-OCT, *en-face*, and 30-degree transverse axial scans. These scans are not routinely performed during ophthalmological examinations at our institution. The absence of these scans may have resulted in BMO obscuration and an underestimation of the prevalence of folds, deeper hypo-reflective cores (buried drusen), and abnormal BM/RPE angulation^10,12,14^.

In summary, this study identified a comprehensive set of OCT biomarkers to a largely undifferentiated population and has demonstrated that OCT biomarkers may provide diagnostic utility in differentiating papilledema from pseudopapilledema in a clinical setting. This has significant diagnostic value and the findings from this study could reduce the necessity of utilising time-consuming, expensive, and invasive investigations that are typically required to differentiate papilledema from pseudopapilledema.

## Electronic supplementary material

Below is the link to the electronic supplementary material.

Supplementary Material 1

## Data Availability

The data that support the findings of this study are not openly available due to reasons of sensitivity and are available from the corresponding author upon reasonable request. Data are located in controlled access data storage at the University of Leicester and can be made available through a trusted research environment and application process.

## Abbreviations

ONH: Optic nerve head
ICP: Intracranial pressure
ODD: Optic disc drusen
TOD: Tilted optic discs
COD: Crowded optic discs
OCT: Optical coherence tomography
BMO: Bruch’s membrane opening
BM/RPE: Bruch’s membrane/retinal pigment epithelium
RNFL: Retinal nerve fibre layer
PHOMS: Peripapillary hyperreflective ovoid mass-like structures
AUC: Area under curve
ROC: Receiver operating characteristics
