# Supplementary material for: Identifying biomarkers for papilledema and pseudopapilledema
Source: Sci Rep. 2025 Jul 10;15:24847. doi: 10.1038/s41598-025-09778-2 (PMC12246050; doi:10.1038/s41598-025-09778-2)
Supplement: Supplementary file 1 — Supplementary Material 1 [file 41598_2025_9778_MOESM1_ESM.docx]

***Title:*** *Identifying Biomarkers for Papilledema and Pseudopapilledema*

| **Authors:**Rishi Sekhri, MBChB,^1^ Helen J. Kuht, BMedSci, PhD,^1^Zhanhan Tu, PhD,^1^ Gail DE Maconachie, BMedSci, PhD,^1,2^ Riddhi Shenoy, MBChB,^1^ Esha Prakash, MBChB,^1^ Seema Teli, BMedSci,^1^ Sohaib Rufai, PhD,^1^ Nagini Sarvananthan, MD, FRCOphth,^3^ Tahir Islam, FRCOphth,^3^ Michael Hisaund, BMedSci,^1^ Rebecca J McLean, PhD,^1^ Indranil Choudhuri, FRCOphth,^3^ Joris Dehaene, FRCOphth,^3^ Martin Barnes, FRCOphth,^3^ Irene Gottlob, MD, FRCOphth,^1,4^ Ian DeSilva, MBChB, FRCOphth,^3^ Mervyn G. Thomas PhD, FRCOphth^1, 3, 5^* |
| --- |
| **Affiliations:**   1. Ulverscroft Eye Unit, School of Psychology and Vision Sciences, College of Life Sciences, University of Leicester, Leicester, UK. 2. School of Allied Health Professions, Nursing and Midwifery, Faculty of Health, University of Sheffield, Sheffield, UK. 3. Department of Ophthalmology, Leicester Royal Infirmary, Leicester, University Hospitals of Leicester NHS Trust, Leicester, UK. 4. Department of Neurology, Cooper University Hospital, Camden, NJ, United States. 5. NIHR Leicester Biomedical Research Centre, University Hospitals of Leicester NHS Trust and University of Leicester, Leicester, UK   *Correspondence:  Mervyn G. Thomas, PhD, FRCOphth, Ulverscroft Eye Unit, School of Psychology and Vision Sciences, University of Leicester, UK. E-mail: [mt350@le.ac.uk](mailto:mt350@le.ac.uk). |

**Supplementary Information**

| **Search Terms** | **Count** |
| --- | --- |
| *Papilloedema or Papilledema* | 6111 |
| *Pseudopapilloedema or Pseudopapilledema* | 220 |
| *Optic disc/disk drusen or optic nerve head drusen* | 942 |
| *Tilted optic disc/disk* | 62 |
| *Crowded optic disc/disk* | 31 |
| *Optical coherence tomography or OCT* | 116,296 |
| *Limit to English language* | 79 |

***Supplementary Table S1:*** *Search terms used in literature review and number of results found on OVID Medline.*

| **Biomarker** | **Odds**  **Ratio** | **P value** | **Sensitivity** | | **Specificity** | |
| --- | --- | --- | --- | --- | --- | --- |
| *BM/RPE Angulation (+ve)* | 4.24 | 0.002 | 40.51 | 73.42 | 86.36 | 81.82 |
| *Folds (presence)* | 16.18 | <0.001 | 54.43 |  | 93.18 |  |

***Supplementary Table S2:*** *P values, sensitivity, and specificity are reported for each significant individual biomarker in the tilted optic disc subgroup analysis. Logistic regression odds ratios reported for each biomarker and overall sensitivity and specificity reported. BM/RPE= Bruch’s membrane/retinal pigment epithelium.*

***Supplementary Figure S1:*** *Literature review search results with number of papers identified for each biomarker reported. OCT= Optical Coherence Tomography, RNFL= retinal nerve fibre layer, BMO= Bruch’s membrane opening, PHOMS= peripapillary hyperreflective ovoid mass-like structures, BM/RPE= Bruch’s membrane/retinal pigment epithelium.*
